# Prognostic implication of a novel lactate score correlating with immunotherapeutic responses in pan-cancer

**DOI:** 10.18632/aging.205423

**Published:** 2024-01-09

**Authors:** Ying Deng, Guoqiang Zhu, Xiao Mi, Xiaoyu Jing

**Affiliations:** 1 Department of Pediatrics, West China Second University Hospital, Sichuan University, Chengdu, China; 2 Key Laboratory of Birth Defects and Related Disease of Women and Children, Ministry of Education, Sichuan University, Chengdu, China; 3 Key Laboratory of Bio-Resources and Eco-Environment of Ministry of Education, College of Life Sciences, Sichuan University, Chengdu, China; 4 College of Animal Science and Technology, Northwest A&F University, Shaanxi Key Laboratory of Molecular Biology for Agriculture, Xianyang, China

**Keywords:** lactate, prognosis, LDHA, immunotherapy response

## Abstract

A thorough assessment of lactate-related genes (LRGs) in different types of human cancers is currently lacking. To elucidate the molecular landscape of LRGs, we conducted a comprehensive analysis using genomic, mRNA, and microRNA expression profiles and developed a lactate score model using the least absolute shrinkage and selection operator (LASSO) algorithm. We found that our lactate score could be a prognostic marker instead of *LDHA* for several cancer patients who possess high-frequency variants in LRGs. The lactate score also demonstrated an association with CD8+ T cells infiltration in multiple cancer types. Furthermore, our findings indicate that the lactate score holds promise as a potential biomarker for immunotherapy in patients with bladder cancer (BLCA) and skin cutaneous melanoma (SKCM). Among the seventeen genes of the lactate score model, *PDP1* showed the strongest positive correlation with lactate score and the potential as a standalone biomarker for prognosis. In general, our study has yielded crucial insights into the potential application of the lactate score as a predictive biomarker for both survival outcomes and the response to immunotherapy. By recognizing the prognostic significance of lactate metabolism, we open avenues for further investigations aimed at harnessing the therapeutic potential of lactate.

## INTRODUCTION

Lactate, once considered a mere byproduct of glycolysis, is now acknowledged as a fundamental carbon source for cellular metabolism, influencing various aspects of carcinogenesis, immune evasion, angiogenesis, metastasis, and therapeutic response [1–3]. The metabolic phenomenon observed in cancer cells, where they predominantly utilize glucose as their primary energy source and produce significant amounts of lactate even in the presence of adequate oxygen, is commonly known as aerobic glycolysis or the Warburg effect. This term stems from Otto Warburg’s pioneering work on the metabolic characteristics of cancer cells [4]. During aerobic glycolysis and glutaminolysis, substantial quantities of lactic acid and hydrogen ions are released into the extracellular space, resulting in the acidification of the tumor microenvironment and the establishment of a reversed pH gradient [5, 6]. Consequently, lactate plays a pivotal role in both tumor development and the remodeling of the tumor microenvironment. Lactate dehydrogenases (LDHs) are a group of metabolic enzymes responsible for the reversible conversion of pyruvate to lactate. Two isoforms, *LDHA* and *LDHB*, exist, each exhibiting a dual role in cancer metabolism [7]. The expression levels of *LDHA* are regulated by several factors, including *HIF1α*, *MYC*, and *p53*, and are associated with epithelial-to-mesenchymal transition, angiogenesis, and increased tumor cell invasion [8]. Conversely, the downregulation or loss of *LDHB* expression correlates with high proliferation, enhanced invasion, and poor survival [9, 10]. Remarkably, lactate has also been implicated in resistance to tyrosine kinase inhibitor therapies, such as the epidermal growth factor receptor inhibitor erlotinib [11]. Additionally, high lactate concentrations have been linked to radiotherapy resistance in nude mice xenografted with human head and neck cutaneous squamous cell carcinoma cell lines [12]. Collectively, these findings provide compelling evidence for the critical role of lactate in tumors, acting as both a metabolic fuel and a signaling molecule.

Lactate levels in the extracellular environment can be detected by cancer cells and several immune cell types, including T cells, natural killing (NK) cells, dendritic (DC) cells, and macrophages, triggering intracellular signaling that regulates cellular behaviors and functions within the tumor microenvironment [2]. Notably, an extracellular pH range of 6.0–6.5 has been found to induce anergy in CD8+ T cells, resulting in reduced cytolytic activity and cytokine production [13]. Furthermore, extracellular acidification suppresses the anti-tumoral activity of NK cells through mTOR inhibition [14]. Additionally, lactate exerts a positive influence on the metabolic profile of regulatory T (Treg) cells, promoting their survival and immunosuppressive functions [15]. Moreover, lactate plays a role in promoting the polarization of alternatively activated macrophages with an M2-like phenotype, contributing to angiogenesis, tissue remodeling, tumor growth, and invasion [16]. In recent years, immune checkpoint inhibitors (ICIs) have demonstrated significant improvements in patient outcomes and have become first-line therapies for several cancer types [17]. However, the response rate to ICIs as monotherapy is limited, with only 20% to 30% of patients exhibiting positive responses [18, 19]. Nonetheless, the relationship between lactate levels and immunotherapy response across different cancer types remains largely unexplored.

The crucial role of lactate in both tumorigenesis and tumor immunity necessitates a comprehensive investigation of lactate levels across different cancer types. To address the gap of a pan-cancer analysis, we embarked on a study utilizing publicly available databases, including the Cancer Genome Atlas (TCGA) and Gene Expression Omnibus (GEO). Our primary objectives were to construct and validate a prognostic model encompassing a wide range of cancers based on lactate-related genes (LRGs). Furthermore, we aimed to explore the potential associations between this model and survival outcome, immune infiltration as well as the response to immunotherapy.

Given the pivotal role of lactate in both tumorigenesis and tumor immunity, it is imperative to thoroughly investigate lactate levels across different types of cancer. However, such investigations have been lacking in the scientific literature. To address this gap, our study was designed to utilize publicly available databases, such as TCGA and GEO. We constructed and validated a prognostic model based on lactate-related genes that encompasses multiple cancer types. Moreover, we sought to explore the correlation between our lactate score model and immune infiltration, as well as its potential implications for immunotherapy response. By adopting this approach, we aim to enhance our understanding of the significant role of lactate in cancer biology and pave the way for clinical applications in the management of cancer patients.

## MATERIALS AND METHODS

### Data acquisition

The work has been reported in line with the REMARK criteria [20]. The clinical and mRNA expression data of 33 cancer types involving 10,251 patients from The Cancer Genome Atlas (TCGA) was downloaded from https://xenabrowser.net/datapages/. The detailed information of the used datasets is displayed in Supplementary Table 1. Out of the 33 cancer types, all had mRNA and microRNA profiles, and 24 cancer types had mutation information. Microarray datasets of GSE14764 and GSE140082, involving 456 patients with ovarian cancer, were downloaded from the Gene Expression Omnibus database (GEO, https://www.ncbi.nlm.nih.gov/geo/) [21, 22]. The results of the analysis of one single-cell dataset of ovarian cancer, GSE151214, were obtained through an online website called Tumor Immune Single-cell Hub 2 (TISCH2, http://tisch.comp-genomics.org/home/) to study the distribution in cell subpopulations [23]. The immunotherapy response datasets involving 253 patients were downloaded from GSE91061, IMvigor210 and PRJEB23709 [24–26].

### Depiction of the lactate-related genes

A total of 230 genes were identified as Lactate-related Genes (LRGs) and acquired from the Molecular Signatures Database (MSigDB; https://www.gsea-msigdb.org) according to five pathways, namely GOBP Lactate Metabolic Process, HP Abnormal Lactate Dehydrogenase Levels, HP Increased CSF lactate, HP Increased Serum Lactate, HP Increased Circulating Lactate Dehydrogenase Concentration. The characteristics of the lactate-related genes were depicted in the 24 cancer types from the TCGA cohort. The somatic single-nucleotide variation of these genes was calculated via R package “maftools” based on sequencing results [27]. The gene-level CNV information was extracted from the sequencing results and utilized for calculation of amplification and deletion frequencies. The oncoprint of the variants was done using the R package ComplexHeatmap [28]. miRNA prediction for PDP1 using the R package multiMiR, upset map using the R package UpSetR [29, 30].

### Evaluation of lactate score

We employed the two algorithms to build Cox proportional-hazards regression model, namely random forest (RF) and least absolute shrinkage and selection operator (LASSO). The models of lactate scores were trained and validated in TCGA-OV patients by R package “randomForestSRC” and “glmnet” were employed [31–33]. When performing the estimation of RF-built models, the candidates associated with overall survival were selected and then put into the machine learning to get the different importance for the models. Then the twenty most important genes were pick for the training of the RF models using Cox proportional-hazards regression. When performing the estimation of LASSO-built models, a total of 230 LRGs were put into the model training and the penalty parameter was selected by cross-validation to avoid the overfitting. The models with highest concordance(c)-index were chosen as the best RF model and the best LASSO model. The receiver operating characteristic (ROC) curves of the two models in predicting 5-year survival of ovarian cancer were implemented by R package “survival ROC”. The corresponding area under curves (AUC) of the two models were compared for the performances of the model building methods.

With the signature constructed by LASSO Cox regression, samples were assigned with lactate score and divided into high-, middle- and low lactate score groups according to the tertiles of lactate score. Kaplan-Meier curves were carried out to compare the survival time differences by the “survival” and “survminer” R package [34, 35]. The 5-year ROC curves and the ROC curves were compared to predict the survival status with the “timeROC” R packages [36]. Besides, ROCs and AUCs of the signature illustrating its performance in predicting 1-, 3-, and 5-year survival were presented for further verification. Validation by external datasets were carried in GSE4764 and GSE140082 cohorts, with the locked model and redefined cut-off values by the tertiles of lactate score.

### Drug sensitivity prediction and immune infiltration

The GDSC2 database contains IC50 and transcriptomic data for 167 drug-treated cell lines using the R package oncoPredict, which predicts drug IC50 for each patient of TCGA-OV based on transcriptomic data [37]. Pearson correlation was calculated using the R package Hmisc, the heatmap using the R package pheatmap, scatter plot and boxplot using the R package ggpubr [38–40]. The codes of cibersort absolute were used to estimate the infiltration levels of immune cells [41, 42]. The infiltration levels were compared among high, middle, and low lactate score groups. We further investigated the correlation between lactate score and response to immunotherapy using eleven datasets with immunotherapy response.

### Statistical analysis

All statistical analyses were performed using R language (https://www.r-project.org/). Wilcox test was used to compare continuous variables between two groups. Pearson coefficient was calculated to measure the correlation between two continuous variables. Detail parameters were described in the corresponding analysis. P-value <0.05 was considered to be statistically significant if not otherwise stated.

## RESULTS

### The genetic landscape of LRGs in pan-cancer

**Figure 1:**
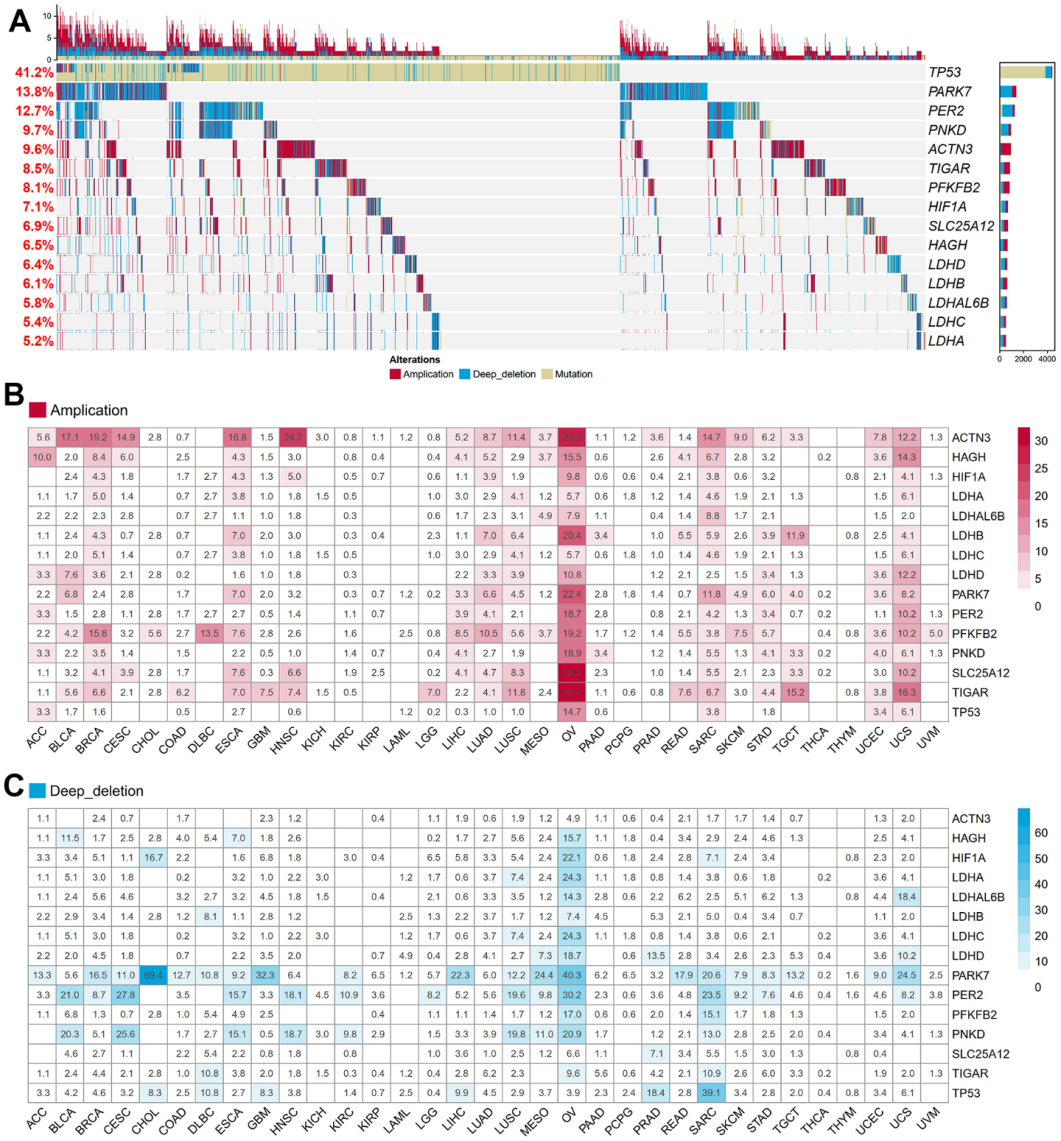
**Genetic and transcriptional alterations of LRGs in 33 cancers.** (**A**) The frequencies of total genetic variations for 15 LRGs from the pathway of GO_BP Lactate Metabolic Process in pan-cancer. Red bars mean copy number amplification, blue bars mean copy number deep deletion, yellow bars mean gene mutation. (**B**) The frequency of copy number amplification for 15 LRGs in each cancer. The numbers in the boxes refer to the percentage of patients with any gene copy number amplification variant compared to all patients in each cancer. (**C**) The frequency of copy number deep deletion for 15 LRGs in each cancer. The numbers in the boxes refer to the percentage of patients with any gene copy number amplification variant compared to all patients in each cancer. LRGs: lactate-related genes; GO_BP: gene ontology biological process.

After excluding a microRNA, we collected a total of fifteen lactate-related genes (LRGs) within the GOBP Lactate Metabolic Process pathway. The oncoprint analysis visually represents the mutation, amplification, and deletion frequencies of these fifteen genes. Among them, *TP53* exhibited the highest variation rate at 41.2%, followed by *PARK7* at 13.8% and *PER2* at 12.7%. Conversely, *LDHA*, *LDHC*, *LDHAL6B*, and *LDHB* displayed the lowest variation frequencies, ranging from 5.2% to 6.1% (Figure 1A). Notably, most LRGs demonstrated variations in gene amplification and deletion, with the exception of *TP53*. To further investigate this, we examined the distribution of copy number variations (CNVs), including amplifications and deletions, across thirty-three different cancers. Interestingly, the frequency of CNVs varied significantly among the different cancer types (Figure 1B, 1C). Kidney chromophobe (KICH), kidney renal clear cell carcinoma (KIRC), kidney renal papillary cell carcinoma (KIRP), acute myeloid leukemia (LAML), pheochromocytoma and paraganglioma (PCPG), prostate adenocarcinoma (PRAD), thyroid carcinoma (THCA), and thymoma (THYM) exhibited lower rates of amplification or deletion events. Conversely, ovarian cancer (OV), breast invasive carcinoma (BRCA), lung squamous cell carcinoma (LUSC), sarcoma (SARC), uterine carcinosarcoma (UCS), and esophageal carcinoma (ESCA) showed a higher frequency of copy number variants in lactate-related genes. These variations can potentially lead to significant abnormalities in mRNA and protein expression, indicating the crucial regulatory role of lactate in these particular cancer types.

### Survival analysis of *LDHA* copy number variation (CNV)

**Figure 2:**
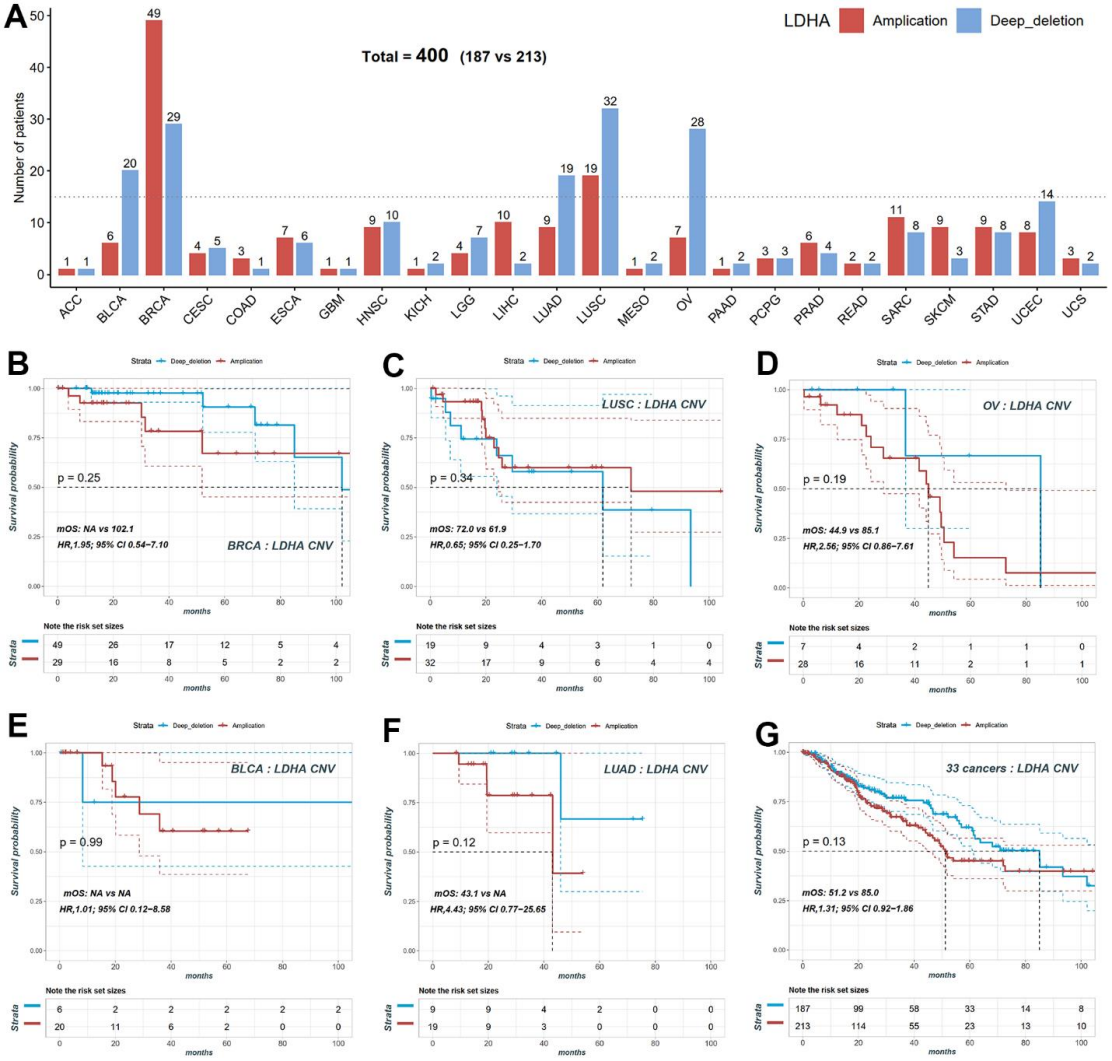
**Copy number variation (CNV) distribution of LDHA across cancers.** (**A**) The frequencies of *LDHA* copy number amplification and deletion across cancers. Red bars mean copy number amplification, blue bars mean copy number deep deletion. (**B**) The KM plot of patients with *LDHA* copy number amplification and deep deletion in BRCA. (**C**) The KM plot of patients with *LDHA* copy number amplification and deletion in LUSC. (**D**) The KM plot of patients with *LDHA* copy number amplification and deletion in OV. (**E**) The KM plot of patients with *LDHA* copy number amplification and deletion in BLCA. (**F**) The KM plot of patients with *LDHA* copy number amplification and deletion in LUAD. (**G**) The KM plot of *LDHA* copy number amplification and deletion all patients. *LDHA*: lactate dehydrogenase A; KM: Kaplan-Meier curves; BRCA: breast invasive carcinoma; LUSC: lung squamous cell carcinoma; OV: ovarian cancer; BLCA: bladder urothelial carcinoma; LUAD: lung adenocarcinoma.

In our study, we focused on lactate dehydrogenase A (*LDHA*), the primary metabolic enzyme responsible for the conversion of pyruvate to lactate along with NAD+. To investigate the impact of LDHA copy number variation (CNV) on patient prognosis, we performed CNV and survival analyses across twenty-four different cancer types using data from the TCGA project. We observed variations in *LDHA* CNV rates among patients with different tumor types. To further analyze the association between *LDHA* CNV and patient prognosis, we examined the number of patients with *LDHA* amplification or deletion in each cancer type (Figure 2A). Among the cancer types, BRCA exhibited the highest number of *LDHA* CNV variants, with forty-nine patients showing *LDHA* amplification and twenty-nine patients displaying *LDHA* deletion (Figure 2A, 2B). Other cancer types such as LUSC, OV, bladder urothelial carcinoma (BLCA), and lung adenocarcinoma (LUAD) also had a significant number of patients with *LDHA* CNV variants (Figure 2A, 2C–2F). To assess the impact of *LDHA* CNV on patient survival, we categorized patients into amplification and deletion groups in five cancer types that had sufficient sample sizes. However, our analysis did not reveal a significant association between *LDHA* CNV and overall survival (OS) in these cancer types (p > 0.05, Figure 2G). These results indicate that *LDHA* CNV status alone may not be suitable for prognostic prediction in these specific cancers.

### Survival analysis of *LDHA* mRNA

**Figure 3:**
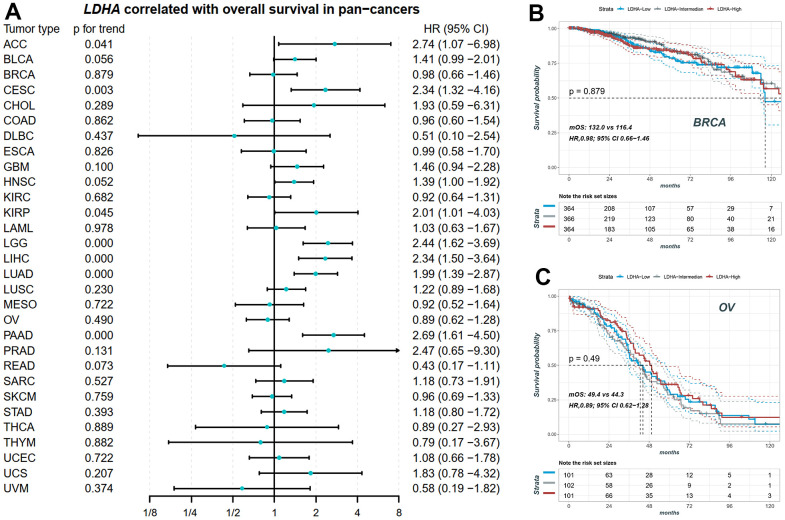
**The prognostic value of *LDHA* in pan-cancers.** (**A**) The expression of *LDHA* did not significantly correlate with overall survival in some cancers with high-frequency mutations in LRGs. (**B**) The KM plot of patients with high, middle and low *LDHA* in BRCA. (**C**) The KM plot of patients with high, middle and low *LDHA* in OV. In each cancer patients were equally divided into three groups of high, middle and low according to their LDHA expression. BRCA: breast invasive carcinoma; OV: ovarian cancer.

We performed Cox correlation analysis to evaluate the relationship between *LDHA* expression levels and OS in various cancer types. The results, presented in the forest plot (Figure 3A), demonstrated that *LDHA* mRNA levels could serve as a prognostic molecular marker in patients with adrenocortical carcinoma (ACC), cervical squamous cell carcinoma and endocervical adenocarcinoma (CESC), Brain lower grade glioma (LGG), LIHC, LUAD, and pancreatic adenocarcinoma (PAAD). However, in cancers with high copy number variants of lactate-related genes (BRCA, OV, LUSC, BLCA, as shown in Figures 1, 2), *LDHA* expression was not associated with survival prognosis. For instance, both BRCA and OV showed log-rank p-values greater than 0.05 (Figure 3B, 3C), indicating that individual *LDHA* gene expression alone may not be suitable for prognostic prediction in these specific cancers.

### Construction of the lactate score in ovarian cancer

**Figure 4:**
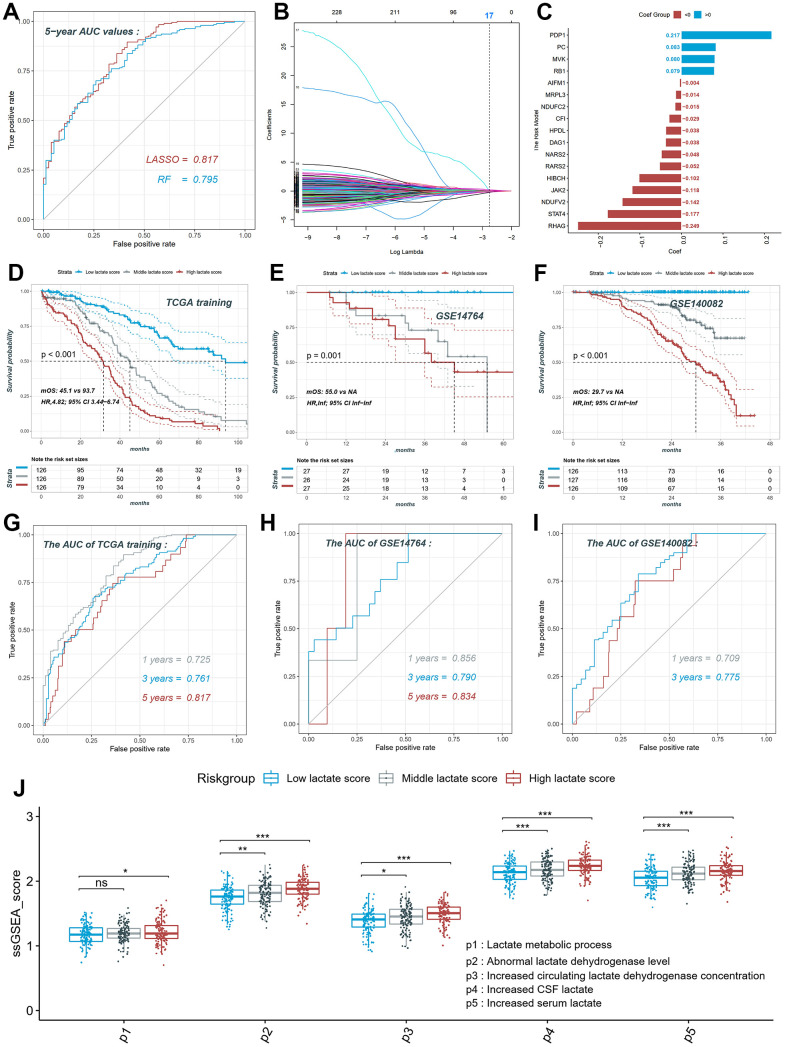
**Construction and validation of the lactate score in ovarian cancer.** (**A**) ROC curve of different models constructed by two algorithms LASSO and RF. (**B**) LASSO coefficient profiles of the 230 LRGs, which come from the pathways named lactate metabolic process, abnormal lactate dehydrogenase levels, increased CSF lactate, increased serum lactate, increased circulating lactate dehydrogenase concentration. (**C**) The risk model consists of 17 genes and their coefficient. (**D**) The KM plot of high, middle and low lactate score patients in training set. (**E**) The KM plot of high, middle and low lactate score patients in GSE14764 dataset. (**F**) The KM plot of high, middle and low lactate score patients in GSE140082 dataset. (**G**) ROC curve of the risk model in training set. (**H**) ROC curve of the risk model in GSE14764 dataset; (**I**) ROC curve of the risk model in GSE140082 dataset. ROC: receiver operating characteristic; LASSO: least absolute shrinkage and selection operator; RF: random forest. LRGs: lactate-related genes.

Using lactate-related genes in five signaling pathways, we utilized TCGA-OV as the training set to compare the performance of two different algorithms in constructing prognostic models based on lactate score. Figure 4A illustrates that the model generated by the LASSO algorithm (AUC=0.817) outperformed the random forest model (AUC=0.795) in predicting the 5-year survival rate of ovarian cancer patients. Therefore, we selected the LASSO model as the final computational model for the lactate score, which comprised the mRNA expression levels of 17 genes (Figure 4B, 4C). The calculation of the lactate score was determined as follows: Lactate score = (PDP1 * 0.217) + (PC * 0.083) + (MVK * 0.080) + (RB1 * 0.079) + (AIFM1 * -0.004) + (MRPL3 * -0.014) + (NDUFC2 * -0.015) + (CF1 * -0.029) + (HPDL * -0.038) + (DAG1 * -0.038) + (NARS2 * -0.048) + (RARS2 * -0.052) + (HIBCH * -0.102) + (JAK2 * -0.118) + (NDUFV2 * -0.142) + (STAT4 * -0.177) + (RHAG * -0.249). Patients in the training set (TCGA-OV) and validation sets (GSE14764 and GSE140082) were categorized into high, middle, and low groups based on the triple quantile of the lactate score (Figure 4D–4F). In the TCGA training group, patients with high lactate scores exhibited significantly worse overall survival prognosis compared to patients with low lactate scores (log-rank p for trend <0.001; HR, 4.82; 95% CI, 3.44-6.74; median overall survival, mOS, 45.1 vs 93.7; Figure 4D). These findings were consistent with the results obtained from the validation sets GSE14764 and GSE140082 (Figure 4E, 4F). The ROC curves of the training set demonstrated AUC values of 0.725, 0.761, and 0.817 for predicting the 1-, 3-, and 5-year survival rates of ovarian cancer patients, respectively (Figure 4G). The AUC values for predicting the 1-, 3-, and 5-year survival rates in the GSE14764 validation group were 0.856, 0.790, and 0.834, respectively, while for the GSE140082 validation group, the AUC values were 0.709 and 0.775 for predicting the 1- and 3-year survival rates, respectively (Figure 4H, 4I). Using the single sample gene set enrichment analysis (ssGSEA), we scored the activity of each of the five lactate-related pathways in TCGA-OV patients and observed significant differences in activity scoring among the high, middle, and low patient groups based on the lactate score (Figure 4J). These results collectively indicate the robustness of the novel lactate score in predicting overall survival.

### Survival analysis of the lactate score in pan-cancer

**Figure 5:**
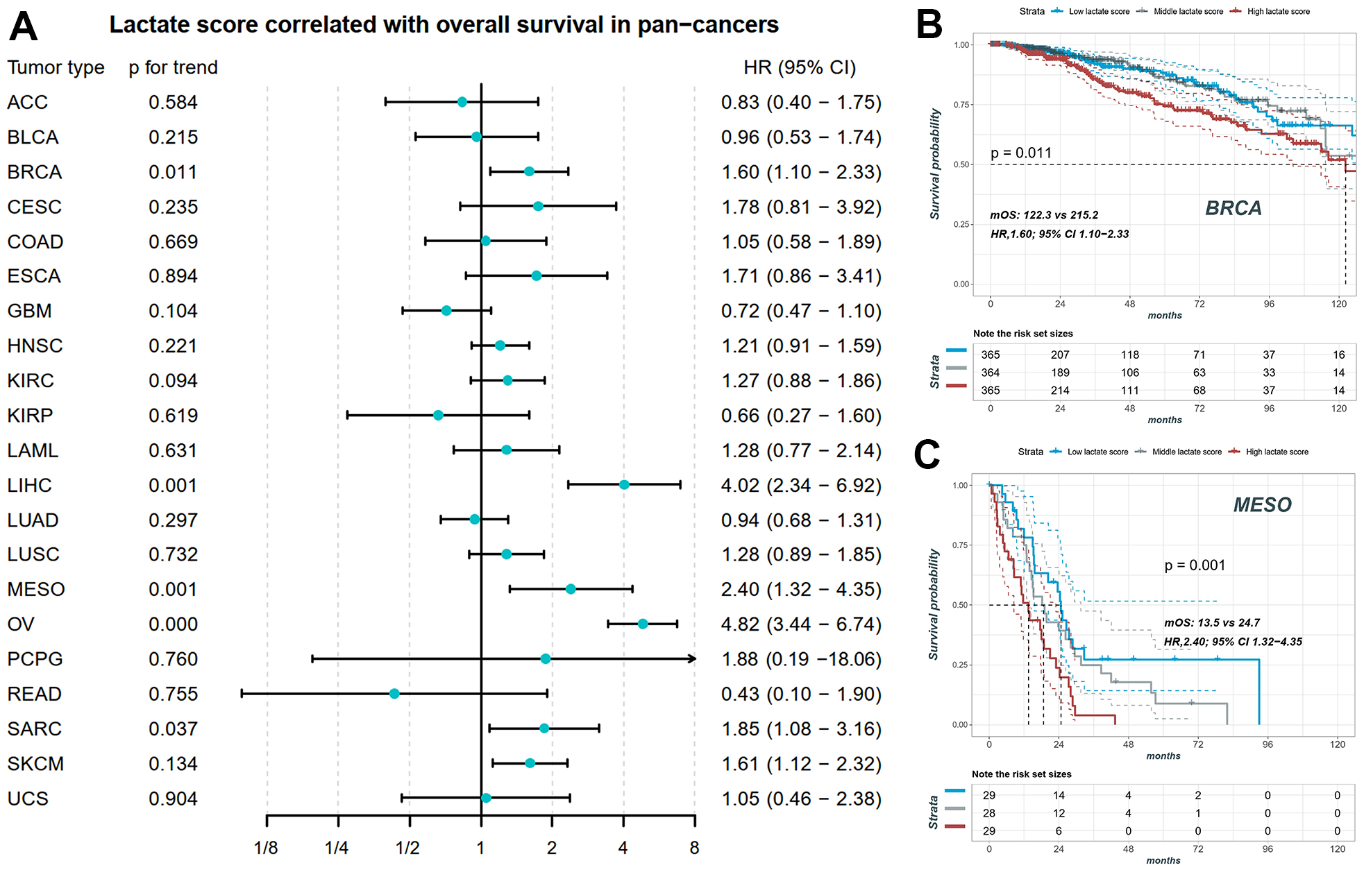
**The prognostic value of lactate score in pan-cancers.** (**A**) Lactate score correlated with overall survival in BRCA, LIHC, MESO, OV, SARC and SKCM. (**B**) The KM plot of patients with high, middle and low lactate score in BRCA. (**C**) The KM plot of patients with high, middle and low lactate score in MESO. BRCA: breast invasive carcinoma; LIHC: liver hepatocellular carcinoma; MESO: mesothelioma; OV: ovarian cancer; SARC: sarcoma; SKCM: skin cutaneous melanoma.

We conducted Cox correlation analysis to assess the relationship between the lactate score and OS across various cancers. Figure 5A demonstrates that the lactate score can serve as a prognostic marker for overall survival in BRCA, LIHC, Mesothelioma (MESO), OV, SARC, and SKCM. Notably, BRCA, OV, and SARC are characterized by high-frequency copy number variants in lactate-related genes. In these cancers, neither copy number variants nor mRNA expression levels of *LHDA* can be utilized as prognostic markers for overall survival. Specifically, in BRCA, patients with high lactate scores exhibited significantly worse overall survival prognosis compared to those with low lactate scores (log-rank p for trend = 0.011; HR, 1.60; 95% CI, 1.10-2.33; mOS, 122.3 vs 215.2; Figure 5B). Similarly, in MESO, patients with high lactate scores demonstrated a significantly poorer overall survival prognosis than those with low lactate scores (log-rank p for trend = 0.001; HR, 2.40; 95% CI, 1.32-4.35; mOS, 13.5 vs 24.7; Figure 5C).

The impact of the sex on the lactate scores and the prognosis in pan-caner has been analyzed in Supplementary Figure 1. In most cancers, there were no significant differences in lactate scores between male and female patient groups (Supplementary Figure 1A–1Z except for 1B, 1I, 1O, 1Q). And only in bladder urothelial carcinoma (BLCA), kidney chromophobe (KICH), lung adenocarcinoma (LUAD), and mesothelioma (MESO), there are significantly different lactate scores between male and female patients (Supplementary Figure 1B, 1I, 1O, 1Q). However, there was no significant difference in overall survival between male and female patients in BLCA, KICH, LUAD and MESO (Supplementary Figure 1B, 1I, 1O, 1Q).

The impact of the age on the lactate scores and the prognosis in pan-caner has been analyzed in Supplementary Figure 2. In most cancers, there were no significant differences in lactate scores between patients aged >65 and <65 years old (Supplementary Figure 2A–2G2 except for 2H, 2U, 2B2). And only in esophageal carcinoma (ESCA), pancreatic adenocarcinoma (PAAD), and testicular cancer (TGCT), there are significantly different lactate scores between patients aged >65 and <65 years old (Supplementary Figure 2H, 2U, 2B2). However, there was no significant difference in overall survival between patients aged >65 and <65 years old in ESCA, PAAD and TGCT (Supplementary Figure 2H, 2U, 2B2).

### Association between the lactate score and clinical therapy in pan-cancer

**Figure 6:**
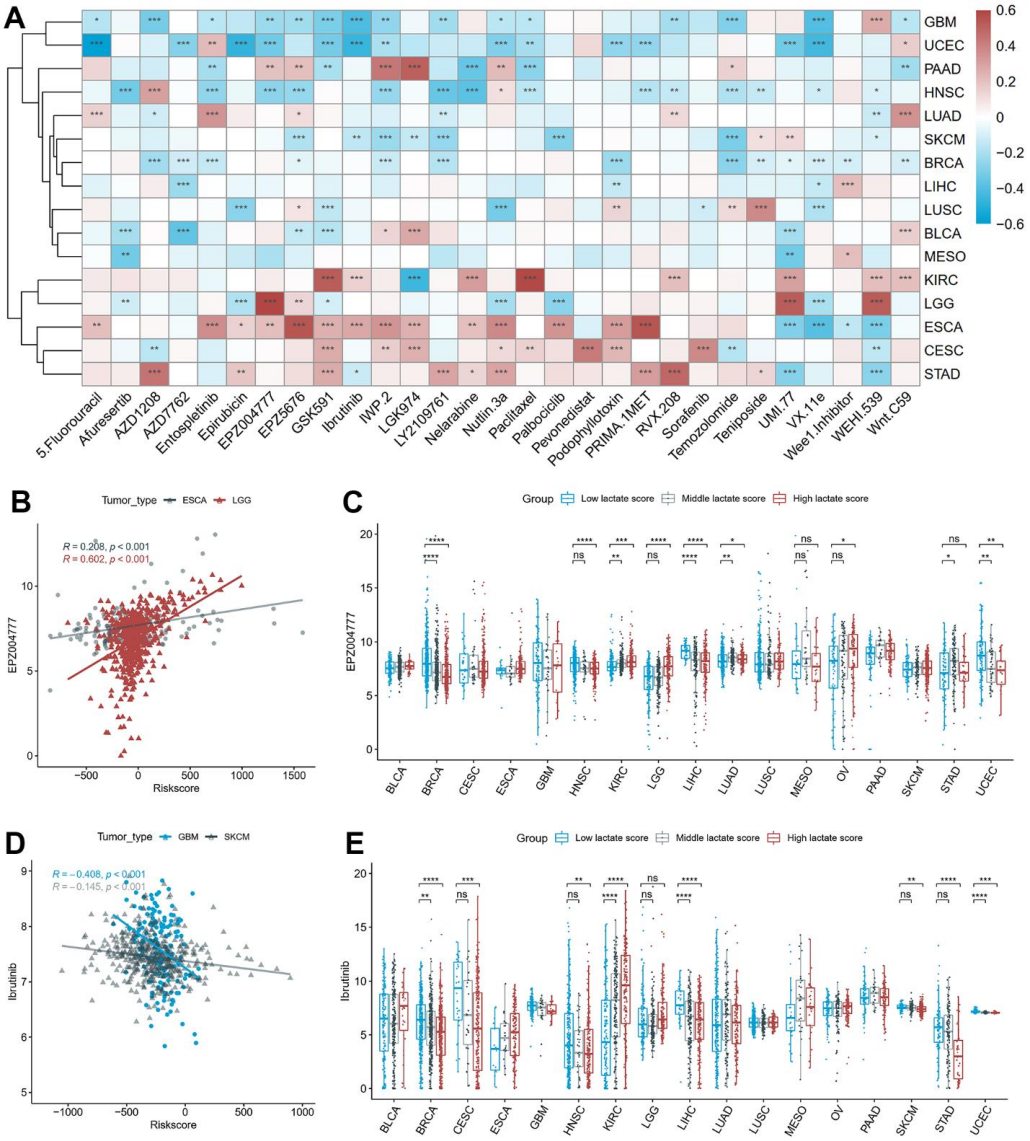
**The correlation between drug sensitivity and lactate scores.** (**A**) The heatmap of the Pearson correlation between lactate scores and IC50 values predicted by oncoPredict. (**B**) The scatter plot of the positive correlation between EPZ004777 and lactate score in ESCA and LGG. (**C**) The differences of EPZ004777 IC50 values between high, middle and low lactate score groups. (**D**) The scatter plot of the negative correlation between Ibrutinib and lactate score in GBM and SKCM. (**E**) The differences of Ibrutinib IC50 values between high, middle and low lactate score groups. ESCA: esophageal carcinoma; LGG: lower grade glioma; GBM: glioblastoma multiforme; SKCM: skin cutaneous melanoma.

We investigated the relationship between the lactate score and drug sensitivities based on the cellular response to drugs obtained from the GDSC database. Among the 16 cancers studied, we identified the two drugs with the highest absolute correlation coefficient for each cancer, resulting in a selection of 29 unique drugs (Figure 6A). EPZ004777 demonstrated the strongest positive correlation with the lactate score, as illustrated in Figure 6B, which depicts its correlation with the lactate score in ESCA and LGG. Our findings suggest that patients with higher lactate scores may exhibit a more favorable response to EPZ004777 in most cancer types, as indicated by lower IC50 values (Figure 6C). Additionally, parallel analysis was conducted on Ibrutinib, which exhibited the strongest negative correlation with the lactate score (Figure 6D, 6E).

### Immune infiltration analysis and prediction of immunotherapy response

**Figure 7:**
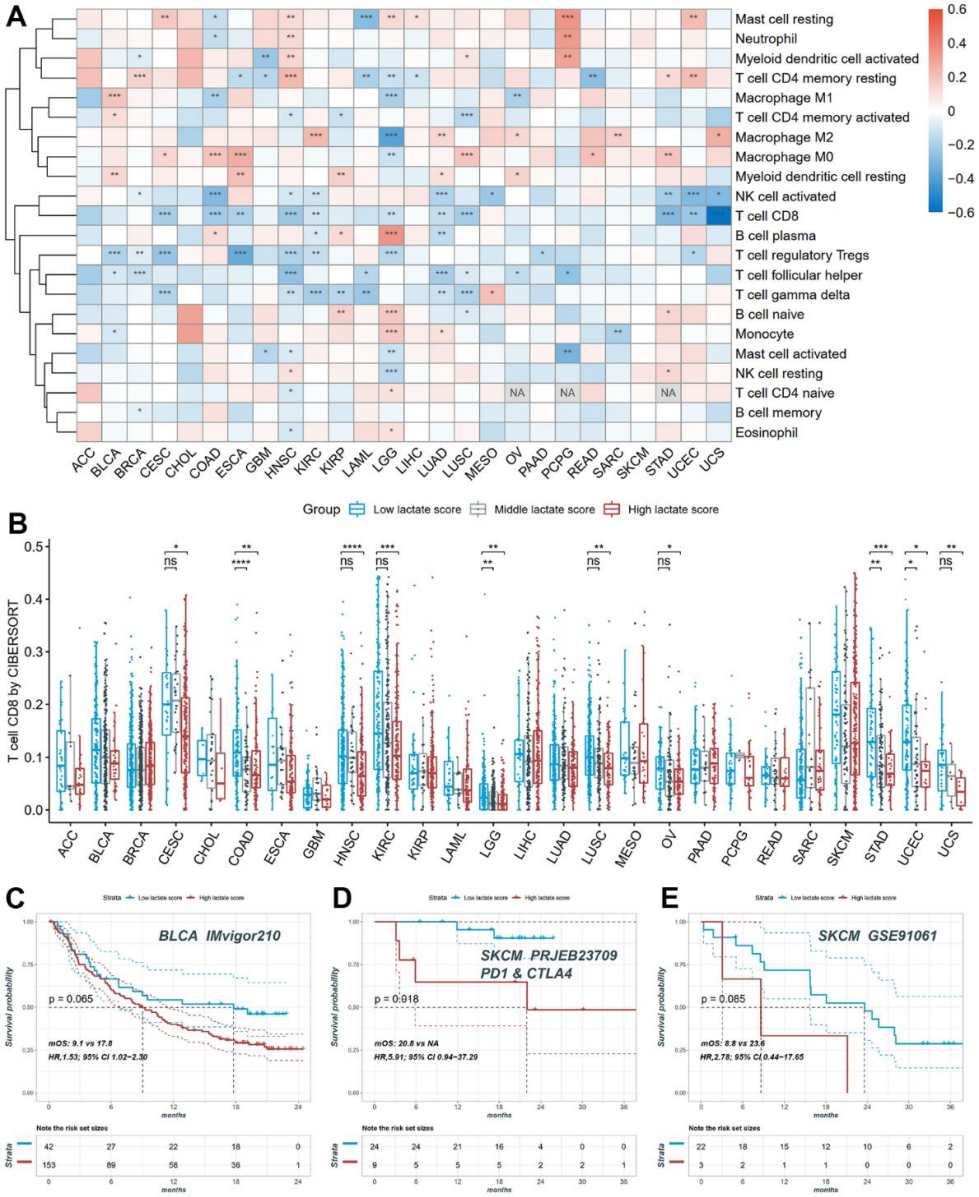
**The distribution of infiltration levels of immune cells and prediction of immunotherapy response.** (**A**) The heatmap of the Pearson correlation between lactate scores and immune cell fractions. (**B**) The differences of CD8+ T cell infiltration between high, middle and low lactate score groups. (**C**) The KM plot of patients in high and low lactate score groups from the BLCA IMvigor210 cohort undergoing immunotherapy. (**D**) The KM plot of patients in high and low lactate score groups from the SKCM PRJEB23709 cohort undergoing immunotherapy. (**E**) The KM plot of patients in high and low lactate score groups from the SKCM GSE91061 cohort undergoing immunotherapy.

We conducted a comprehensive investigation to assess the relationship between the lactate score and the relative fraction of different immune cell types. Pearson correlation analysis was performed to explore this association in each cancer type. Our analysis revealed a significant correlation between lactate scores and immune cell infiltration, as computed by the Absolute CIBERSORT algorithm, in the majority of cancers (Figure 7A). Specifically, in COAD, HNSC, KIRC, LUAD, STAD, UCEC, and UCS, both CD8+ T cells and activated NK cells showed a significant and negative association with lactate scores (Figure 7A). Furthermore, we compared the infiltrations of CD8+ T cells among high, middle, and low lactate score groups across different cancers. Consistently, our results demonstrated higher immune infiltration of CD8+ T cells in the low lactate score group compared to the high and middle lactate score groups in CESC, COAD, HNSC, LGG, LUSC, OV, STAD, UCEC, and UCS (Figure 7B). These findings highlight a notable association between the lactate score and tumor immunity. We also investigated the correlation between the lactate score and the response to immunotherapy in BLCA and SKCM. Remarkably, the successively ranked immunotherapy response rates were higher in the low-score group than in the high lactate score group in the BLCA IMvigor210 cohort (Figure 7C). This trend was consistently observed in the SKCM PRJEB23709 and SKCM GSE91061 cohorts (Figure 7D, 7E). Kaplan-Meier curves demonstrated a significant difference in survival outcomes between the high and low lactate score groups in the SKCM PRJEB23709 cohort, with patients in the low lactate score group exhibiting better survival outcomes (p = 0.018, Figure 7D). These findings suggest that the lactate score may hold potential prognostic value for predicting immunotherapy response.

### Survival and immune infiltration analysis of PDP1

**Figure 8:**
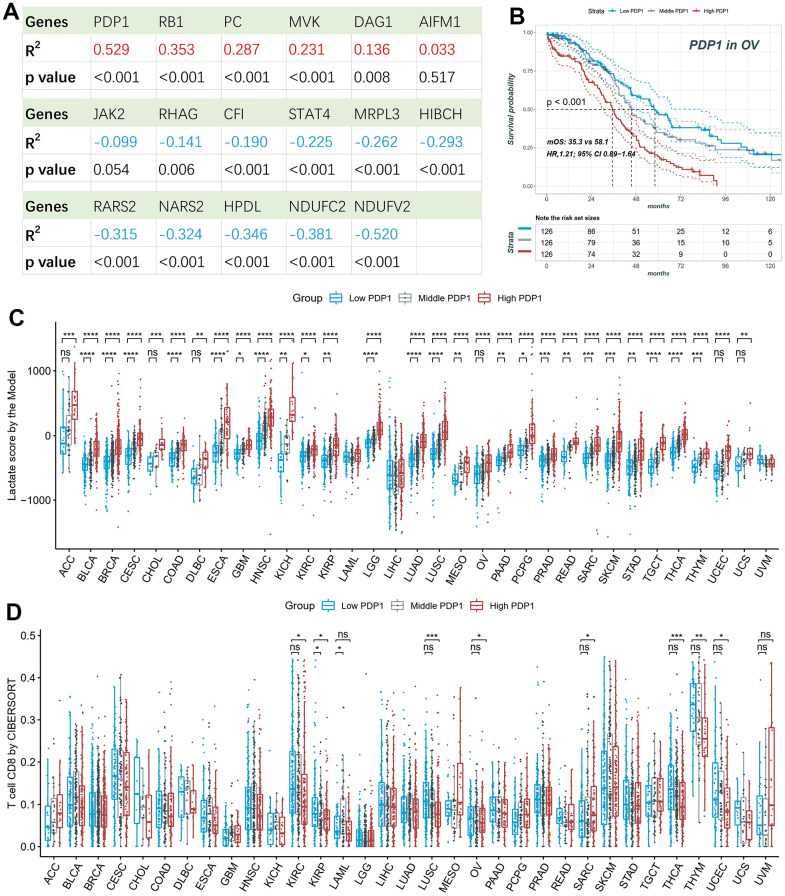
**Survival and immune infiltration analysis of PDP1.** (**A**) The Pearson correlation of the lactate scores and the expression of 17-model genes. (**B**) PDP1, as the highest correlated gene, is associated with overall survival in OV patients. (**C**) The lactate scores are significantly different among high, middle and low *PDP1* expression groups in most cancers. (**D**) The CD8+ T cell infiltrations are significantly different among high, middle and low *PDP1* expression groups.

We performed correlation analysis between the seventeen genes included in the lactate score model and the lactate score in ovarian cancer. Among these genes, *PDP1* exhibited the strongest positive correlation (Figure 8A). Furthermore, Kaplan-Meier analysis confirmed that the expression of *PDP1* was significantly associated with survival outcomes, with patients showing lower expression of *PDP1* tending to have better survival in OV (Figure 8B). Next, we classified the samples into high, middle, and low groups based on the expression of *PDP1*. Interestingly, patients in the high *PDP1* group consistently exhibited higher lactate scores compared to the middle and low *PDP1* groups across multiple cancer types (Figure 8C). Moreover, the low *PDP1* groups demonstrated higher immune infiltration of CD8+ T cells compared to the middle and high *PDP1* groups (Figure 8D).

### Target miRNAs prediction and single-cell transcriptomic analysis

**Figure 9:**
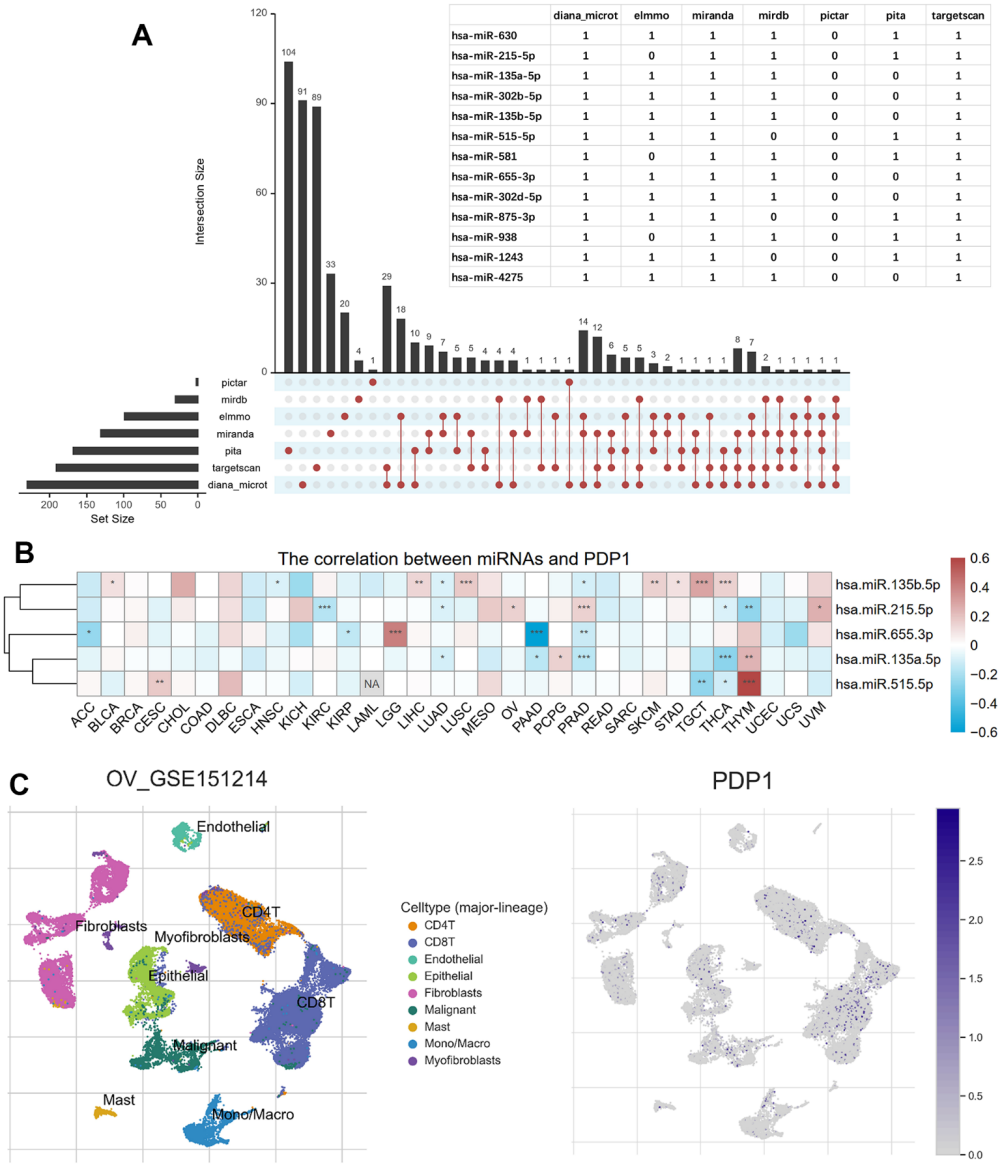
**Predicted *PDP1*-target miRNAs and single-cell transcriptomic analysis.** (**A**) The miRNAs targeting *PDP1* predicted by the seven microRNA-mRNA links databases. And the overlapped miRNAs among more than five databases shown in the table. (**B**) The heatmap of the Pearson correlation between *PDP1* expression and the five miRNAs detected in TCGA. (**C**) The distribution of different cells in ovarian cancer of GSE151214 and the location of *PDP1* expression.

Using seven miRNA target gene prediction databases, namely Pictar, miRDB, ElMMo, Miranda, PITA, TargetScan, and DIANA-microT, a total of 528 target miRNAs were predicted. Among them, 13 miRNAs were predicted by more than five databases, as depicted in Figure 9A. Among these 13 miRNAs, we further investigated the expression data of five miRNAs in TCGA and their correlation with *PDP1* expression in various cancer types. Notably, hsa.miR.655.3p exhibited a positive regulation of *PDP1* in LGG but a negative regulation in PAAD. Similarly, hsa.miR.135b.5p displayed a positive regulation of *PDP1* in LGG but a negative regulation in HNSC and PAAD, while showing a positive regulation in seven other cancer types. These findings highlight both commonalities and heterogeneities in the regulation of *PDP1* among different tumor types (Figure 9B). To gain further insights, we utilized the single-cell sequencing dataset GSE151214 and obtained cell sample annotations from the TISCH database. The analysis revealed that *PDP1* exhibited equalized and ubiquitous expression across various cell types, suggesting its potential as a biomarker (Figure 9C).

## DISCUSSION

In our study, our objective was to explore the landscape of lactate-related genes (LRGs) and their prognostic value, relationship with the tumor microenvironment, and therapeutic responses in pan-cancers. To begin with, we performed a pan-cancer genomic analysis of the GOBP Lactate Metabolic Process pathway and found that copy number variants (CNV) in genes related to lactate metabolism are highly prevalent in cancers such as OV, BRCA, SARC, UCS. But in these cancers, survival analysis of CNV and expression level indicated *LDHA* cannot be utilized for prognostic prediction. Therefore, we conducted a lactate score model consisting of seventeen genes using LASSO algorithm in TCGA and GEO datasets. We found that the lactate score was significantly associated with patient survival, and patients in the low lactate score groups had worse OS in BRCA, LIHC, MESO, OV, SARC, and SKCM. Next, we explored the correlation between the lactate score and immune immunity. We found that lactate score was negatively correlated with CD8+ T cells in almost all tumors and found potential application of lactate score as a biomarker in BLCA and SKCM patients receiving immunotherapy. Interestingly, *PDP1* was found to be a key member in the model and the expression of *PDP1* was associated with survival outcome, implying that the *PDP1* gene alone can be applied as an alternative to the lactate score model with weakened detection performance when all seventeen genes of the lactate score model are restricted. Last we predicted the target miRNAs of *PDP1* and constructed single-cell transcriptomic analysis of *PDP1*.

The Warburg effect represents a fundamental metabolic phenomenon observed in cancer cells, characterized by their preference for utilizing glucose as the primary energy source and producing substantial amounts of lactate, even in the presence of adequate oxygen [4]. Consequently, lactate is released into the extracellular space, leading to an accumulation of lactate within the tumor microenvironment, a factor associated with poor prognoses [43, 44]. Hypoxia, a prevalent feature in most tumors, drives the metabolic reprogramming of cancer cells, resulting in heightened glycolytic activity [45]. Transcription factors such as *HIF-1α* and *c-Myc* play pivotal roles in sustaining elevated lactate levels through diverse mechanisms [46]. Critically, lactate is recognized by cancer cells and various immune cells, including T cells, natural killer cells, dendritic cells, and macrophages, stimulating intracellular signaling pathways that contribute to acidosis, angiogenesis, and immunosuppression [2]. Consequently, lactate assumes a crucial role in tumor development and the remodeling of the tumor microenvironment.

Accumulating evidence supports the critical role of lactate dehydrogenase (LDH) in regulating lactate metabolism through its involvement in the conversion of pyruvate to lactate. Among the LDH isoforms, *LDHA* specifically catalyzes the conversion of pyruvate to lactate, accompanied by the conversion of NADH to NAD+. This metabolic adaptation ensures a continuous supply of “fuel” for cancer cells by reducing the entry of pyruvic acid into the tricarboxylic acid cycle, thereby facilitating tumor growth and invasiveness, even in hypoxic conditions [3, 47]. Notably, previous studies have demonstrated an association between elevated *LDHA* expression and poor survival outcomes in various types of tumors [48], highlighting *LDHA* as a potential prognostic indicator [49]. In this study, we investigated the relationship between CNV of *LDHA* and patient survival across twenty-four different cancer types in TCGA. Surprisingly, our findings revealed no significant correlation between *LDHA* CNV and OS, suggesting that individual *LDHA* CNV alone is insufficient for prognostic prediction. Consequently, we conducted a comprehensive pan-cancer analysis using a lactate score model, which holds promise in predicting prognosis and response to immunotherapy.

The “Warburg effect” represents a characteristic metabolic alteration in cancer cells, characterized by increased glucose consumption and lactate production to support rapid cell proliferation. Lactate levels are upregulated in cancer cells through various mechanisms [46]. Specifically, in cervical cancer, physiological lactate levels can range from 4 mM to 40 mM, whereas normal tissues maintain levels of only 1.8–2 mM [50, 51]. High lactate levels have been associated with poor clinical outcomes in several cancer types [52], and elevated lactate levels have been linked to metastasis in cervical, breast, head, and neck cancers [50, 53, 54]. The accumulation of lactate in the tumor microenvironment alters the metabolic landscape, providing fuel for cancer cells and contributing to acidosis, inflammation, angiogenesis, immunosuppression, and radio resistance [55]. Consequently, tumor lactate, serum lactate, and lactate dehydrogenase (LDH) levels have been recognized as prognostic biomarkers in various cancer types [53, 55, 56]. Given the high prevalence of copy number variants in lactate-related genes in ovarian cancer, we constructed a lactate score model using data from the TCGA-OV cohort. Patients with high lactate scores demonstrated significantly worse overall survival compared to those with low lactate scores, a finding validated in independent datasets (GSE14764 and GSE140082). To explore the prognostic value of the lactate score across multiple cancer types, we performed univariate Cox regression analysis, revealing a significant association between the lactate score and overall survival in BRCA, LIHC, MESO, OV, SARC, and SKCM. Kaplan-Meier analysis further confirmed the prognostic value of the lactate score in BRCA and MESO. These findings highlight the association between the lactate score and survival outcomes, suggesting its potential as a biomarker for prognostic prediction.

Elevated lactate levels in the TME have been shown to exert immunosuppressive effects, inhibiting the antitumor activity of immune cells. This is primarily mediated through the release of H+ ions, resulting in acidification of the TME [57]. Acidic conditions with an extracellular pH of 6.0–6.5 can induce an anergic state in CD8+ T cells, leading to reduced cytolytic activity and cytokine production [13]. Similarly, NK cell function is suppressed under acidic conditions through mTOR inhibition [14]. Lactic acidosis promotes the differentiation of monocytes into dendritic cells with an immunosuppressive phenotype and inhibits the function of M1 macrophages, characterized by decreased expression of IL-6, iNOS, and CCL2 [2, 58]. Furthermore, lactate plays a role in the metabolic profile of regulatory T (Treg) cells, supporting their survival and immunosuppressive functions [15]. Additionally, lactate promotes the polarization of alternatively activated macrophages with an M2-like phenotype, contributing to angiogenesis, tissue remodeling, and facilitating tumor growth and invasion [16]. In our study, we observed a significant correlation between the lactate score and tumor immunity. Specifically, the low lactate score group exhibited significantly higher levels of CD8+ T cell infiltration compared to the high and median lactate score groups. This suggests that a low lactate score, indicative of reduced lactate levels and potentially associated with immune cell activation, may be linked to a more favorable prognosis.

Checkpoint inhibitors, including CTLA-4 and PD-1/PD-L1 inhibitors, have demonstrated significant clinical efficacy in various cancer types. However, the effectiveness of immunotherapeutic approaches is often hampered by the immunosuppressive nature of the tumor microenvironment, which arises, in part, from the metabolic interactions between tumor cells and infiltrating immune cells. Previous studies have suggested that lactate-related signatures are correlated with immune responses and may have implications for immunotherapy outcomes in SKCM and KIRC, given the immune characteristics of the tumor microenvironment [59, 60]. In order to explore the connection between the lactate score and immunotherapy response, we specifically examined the correlation between lactate score and response to immunotherapy in BLCA and SKCM. Our findings indicate that the lactate score is associated with the response to immunotherapy and may potentially serve as a valuable prognostic indicator for predicting immunotherapy response in BLCA and SKCM.

We conducted a more detailed investigation of the seventeen genes comprising the lactate score model and found that *PDP1* exhibited the strongest positive correlation with the lactate score. Furthermore, Kaplan-Meier analysis confirmed that the expression of *PDP1* was significantly associated with patient survival, with lower expression of *PDP1* being indicative of better survival outcomes. *PDP1* is widely expressed in epidermal growth factor activated cells and various malignant human cancer cells. Notably, the functional activity of *PDP1* is hindered by the acetylation of K202, which disrupts its interaction with the substrate *PDHA1*. This disruption is crucial for promoting glycolysis in cancer cells and subsequent tumor growth. Additionally, phosphorylation of Y381 on *PDP1* results in the dissociation of *SIRT3* and facilitates the recruitment of *ACAT1* to the pyruvate dehydrogenase complex. These distinct posttranslational modifications collaboratively contribute to driving the Warburg effect [61]. Our findings further validate the significant role of *PDP1* in the Warburg effect and tumor growth, highlighting its potential as an independent prognostic biomarker.

Our study offers valuable insights into the landscape of lactate-related genes (LRGs) in various cancer types and highlights the potential predictive value of the lactate score in terms of prognosis and immunotherapy response. Nevertheless, it is important to acknowledge the limitations of our study. Firstly, as a retrospective study, the association between the lactate score and survival outcomes would benefit from further validation in prospective clinical studies. Secondly, additional experimental investigations are necessary to elucidate the mechanisms underlying the relationship between lactate and the response to immunotherapy. These limitations present opportunities for future research to enhance our understanding of the clinical implications of lactate in cancer.

In summary, our study offers a comprehensive analysis of lactate-related genes (LRGs) in a broad range of thirty-three cancer types. The results emphasize the prognostic value of the lactate score, its influence on immune infiltration, and its correlation with the response to immunotherapy in diverse cancer types. These findings provide novel insights into the prognostic significance of lactate and open new avenues for investigating the interplay between lactate and the tumor microenvironment, as well as the potential implications for immunotherapy response.

## Supplementary Material

Supplementary Figures

Supplementary Table 1
