# Supplementary material for: Prognostic implication of a novel lactate score correlating with immunotherapeutic responses in pan-cancer
Source: Aging (Albany NY). 2024 Jan 9;16(1):820–43. doi: 10.18632/aging.205423 (PMC10817381; doi:10.18632/aging.205423)
Supplement: Supplementary Figures [file aging-16-205423-s001.pdf]

SUPPLEMENTARY FIGURES

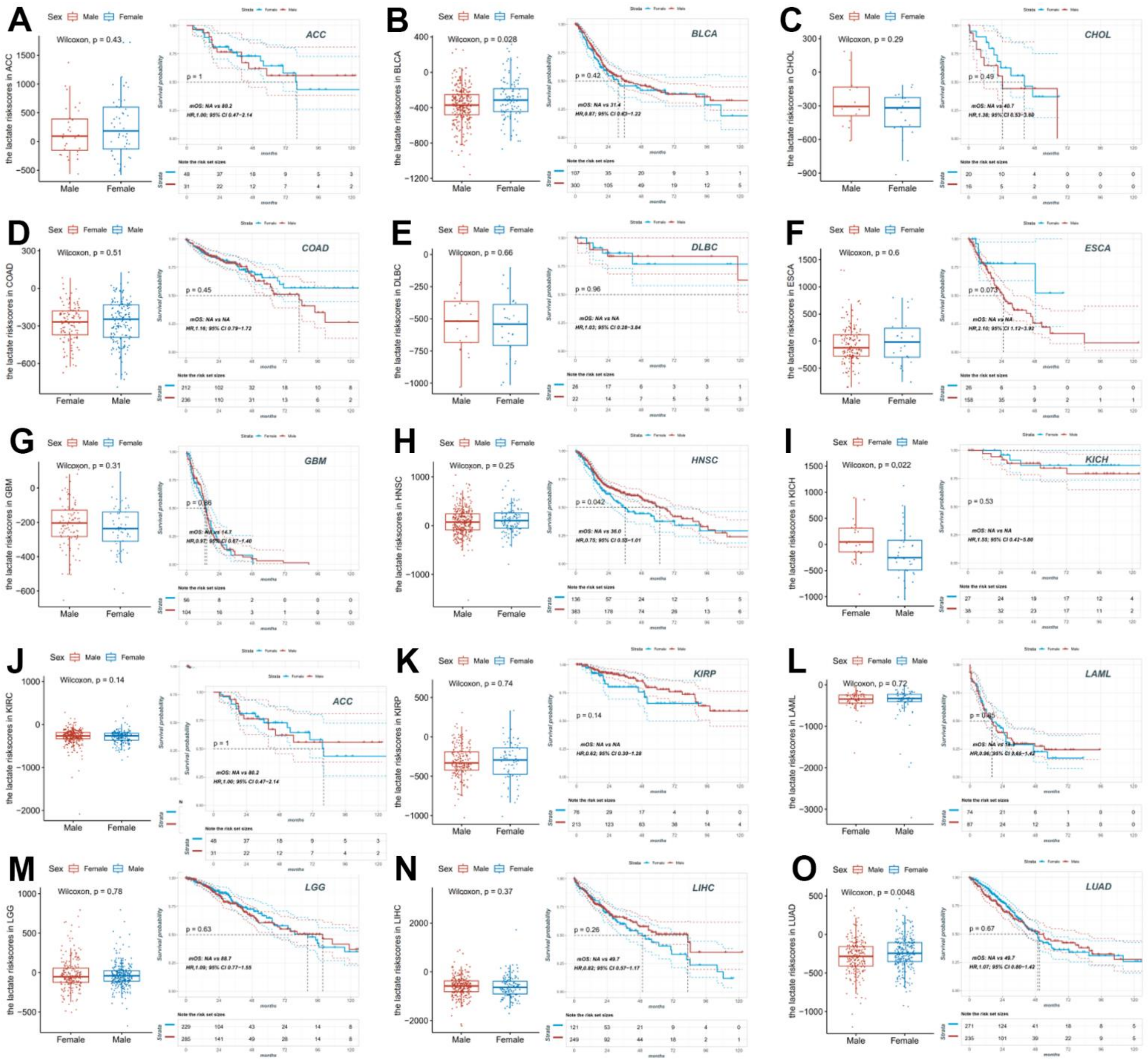

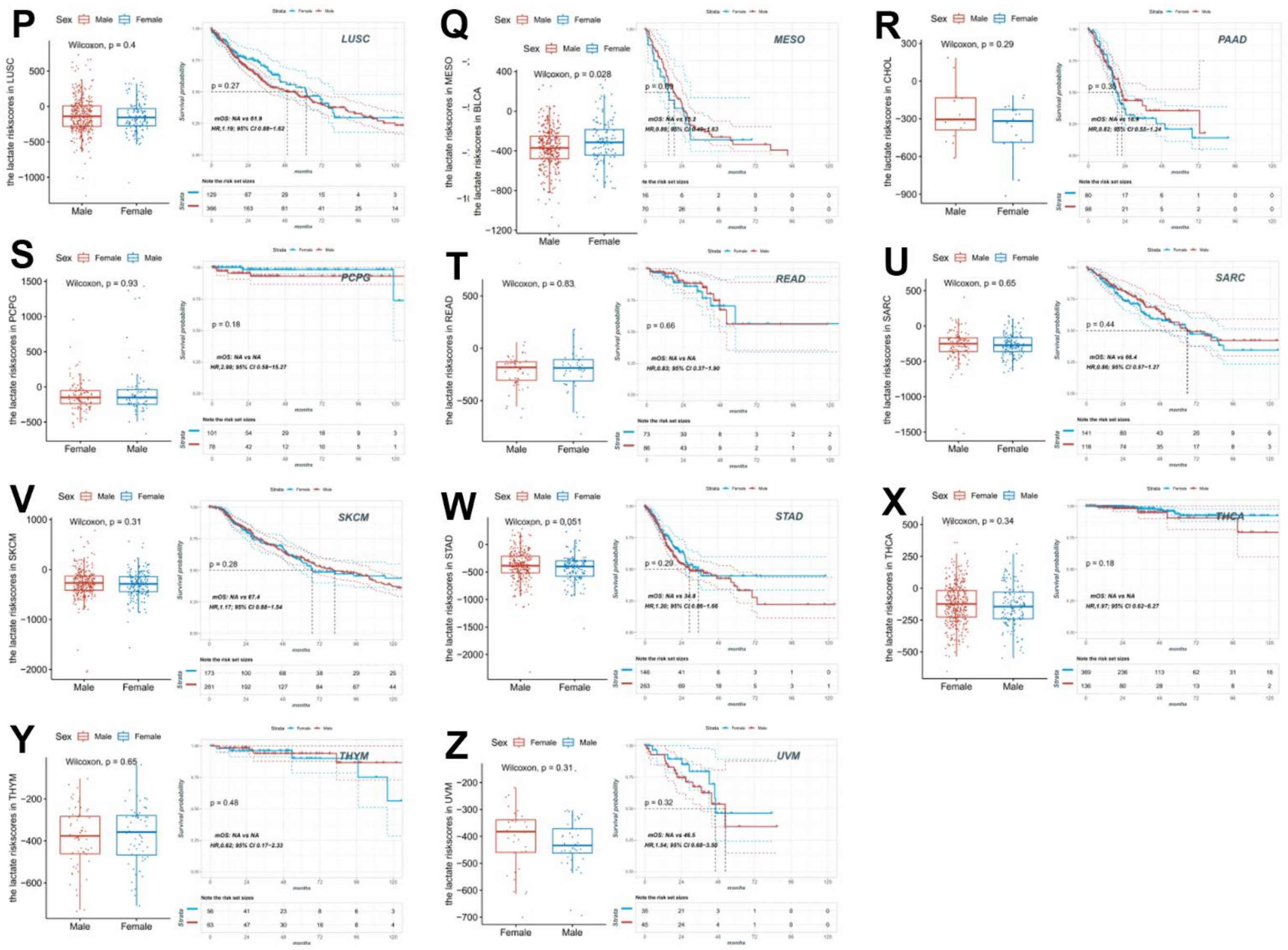

Supplementary Figure 1. The impact of the sex on the lactate scores and the prognosis in pan-caner. The boxplots of lactate scores and the KM plots between male and female patients with different tumors (**A**–**Z**).

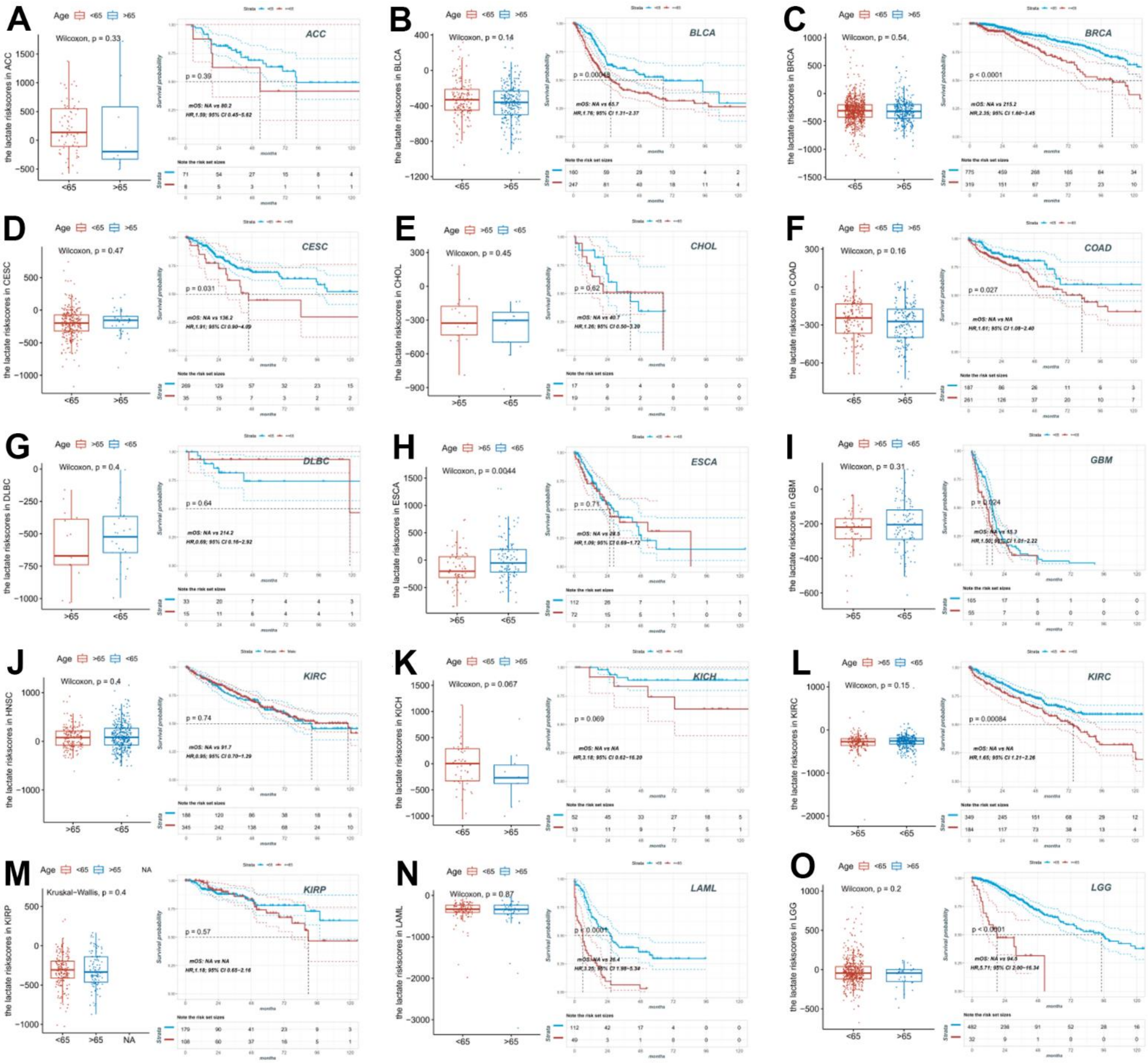
A
Wilcoxon, p = 0.33
ACC
p = 0.39
B
Wilcoxon, p = 0.14
BLCA
C
Wilcoxon, p = 0.54
BRCA
p < 0.0001
D
Wilcoxon, p = 0.47
CESC
p = 0.031
E
Wilcoxon, p = 0.45
CHOL
p = 0.62
F
Wilcoxon, p = 0.16
COAD
p = 0.027
G
Wilcoxon, p = 0.4
DLBC
p = 0.64
H
Wilcoxon, p = 0.0044
ESCA
p = 0.71
I
Wilcoxon, p = 0.31
GBM
J
Wilcoxon, p = 0.4
KIRC
p = 0.74
K
Wilcoxon, p = 0.067
KICH
p = 0.069
L
Wilcoxon, p = 0.15
KIRC
p = 0.00084
M
Kruskal-Wallis, p = 0.4
KIRP
p = 0.57
N
Wilcoxon, p = 0.87
LAML
O
Wilcoxon, p = 0.2
LGG

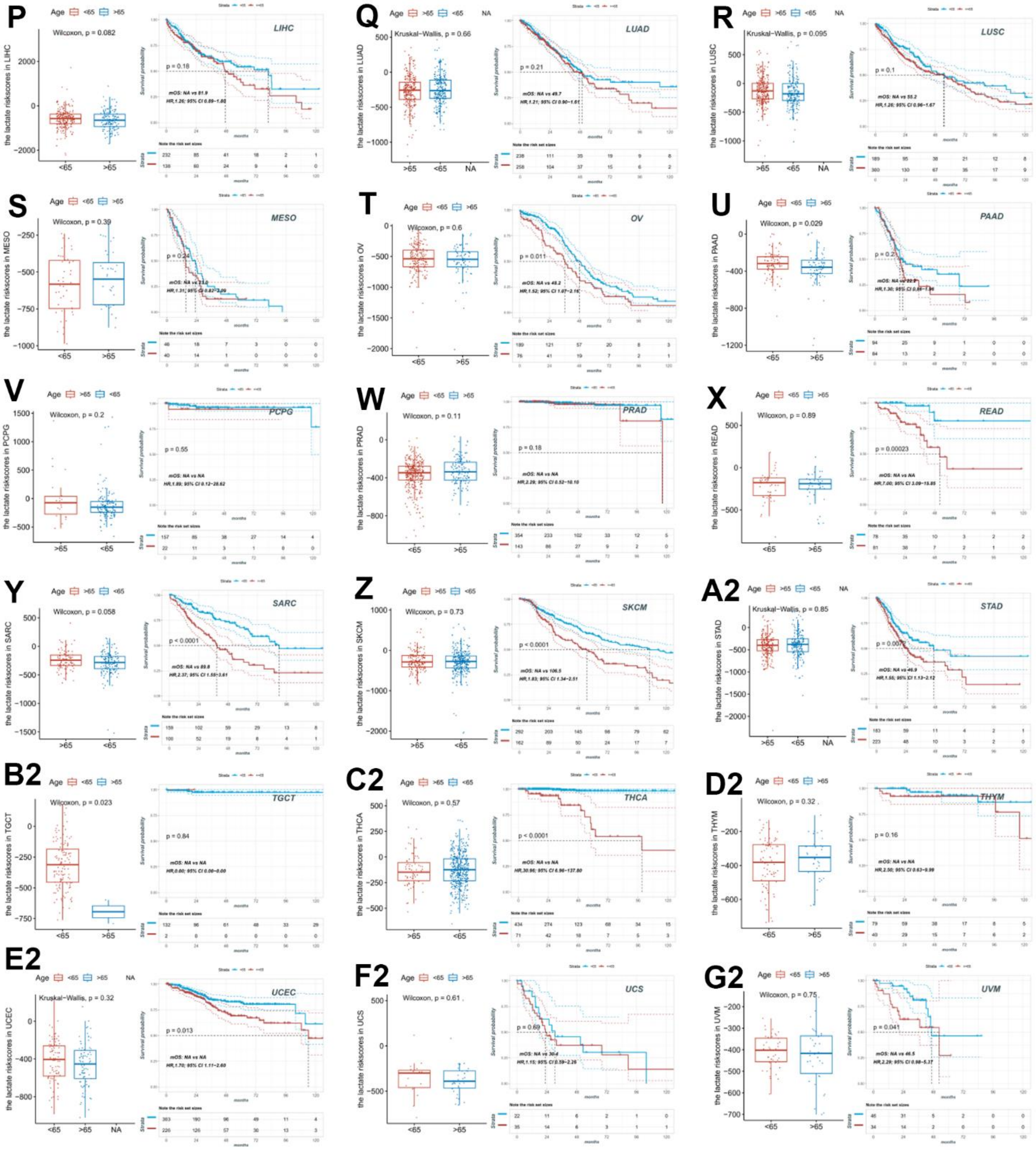

Supplementary Figure 2. The impact of the age on the lactate scores and the prognosis in pan-caner. The boxplots of lactate scores and the KM plots between patients aged >65 and <65 years old with different tumors (**A**–**G2**).
