# Supplementary material for: Prognostic implication of a novel lactate score correlating with immunotherapeutic responses in pan-cancer
Source: Aging (Albany NY). 2024 Jan 9;16(1):820–43. doi: 10.18632/aging.205423 (PMC10817381; doi:10.18632/aging.205423)
Supplement: Supplementary Table 1 [file aging-16-205423-s002.pdf]

SUPPLEMENTARY TABLE

Supplementary Table 1. List of TCGA cancer types.

Study abbreviation	Study name	Tumor	Normal
ACC	Adrenocortical carcinoma	79	3
BLCA	Bladder Urothelial Carcinoma	407	19
BRCA	Breast invasive carcinoma	1101	113
CESC	Cervical squamous cell carcinoma and endocervical adenocarcinoma	306	3
CHOL	Cholangiocarcinoma	36	-
COAD	Colon adenocarcinoma	451	42
DLBC	Lymphoid Neoplasm Diffuse Large B-cell Lymphoma	48	-
ESCA	Esophageal carcinoma	185	11
GBM	Glioblastoma multiforme	168	5
HNSC	Head and Neck squamous cell carcinoma	522	44
KICH	Kidney Chromophobe	65	24
KIRC	Kidney renal clear cell carcinoma	534	72
KIRP	Kidney renal papillary cell carcinoma	290	32
LAML	Acute Myeloid Leukemia	161	-
LGG	Brain Lower Grade Glioma	532	-
LIHC	Liver hepatocellular carcinoma	373	50
LUAD	Lung adenocarcinoma	509	59
LUSC	Lung squamous cell carcinoma	496	50
MESO	Mesothelioma	86	-
OV	Ovarian serous cystadenocarcinoma	308	-
PAAD	Pancreatic adenocarcinoma	179	4
PCPG	Pheochromocytoma and Paraganglioma	184	3
PRAD	Prostate adenocarcinoma	498	52
READ	Rectum adenocarcinoma	161	10
SARC	Sarcoma	263	2
SKCM	Skin Cutaneous Melanoma	457	1
STAD	Stomach adenocarcinoma	412	32
TGCT	Testicular Germ Cell Tumors	139	-
THCA	Thyroid carcinoma	513	58
THYM	Thymoma	119	2
UCEC	Uterine Corpus Endometrial Carcinoma	532	35
UCS	Uterine Carcinosarcoma	57	-
UVM	Uveal Melanoma	80	-
